# Linguistic analysis of plain language summaries and corresponding scientific summaries of Cochrane systematic reviews about oncology interventions

**DOI:** 10.1002/cam4.5825

**Published:** 2023-03-23

**Authors:** Jelena Šuto, Ana Marušić, Ivan Buljan

**Affiliations:** ^1^ Department of Oncology and Radiotherapy Clinical Hospital Centre Split Split Croatia; ^2^ Department of Research in Biomedicine in Health, Center for Evidence‐Based Medicine University of Split School of Medicine Split Croatia; ^3^ Department of Psychology University of Split Faculty of Humanities and Social Sciences Split Croatia

**Keywords:** cancer education, cancer prevention, community outreach, epidemiology and prevention

## Abstract

**Background:**

Cochrane plain language summaries (PLSs) are an important format to present high‐quality healthcare evidence to patients with cancer and their families. They should be written in a way everyone can understand, since they serve as a tool in decision‐making and present a bridge to overcome the gap between the healthcare users and professionals.

**Objective:**

The aim of the study was to assess the language characteristics of PLSs of Cochrane systematic reviews of oncology interventions in comparison with corresponding Cochrane scientific abstracts (SAs).

**Methods:**

In this cross‐sectional study, we included all Cochrane PLSs and SAs of systematic reviews of oncology interventions available in the Cochrane Database of Systematic Reviews. We assessed text readability, measured using the Simple Measure of Gobbledygook (SMOG) index, and the prevalence of words related to different language tones (clout, authenticity, emotions and analytical tones). Two independent assessors categorized the conclusiveness of the efficacy of interventions into nine categories.

**Results:**

The overall median SMOG index for 275 PLSs was 13.0 (95% confidence interval [CI] 12.8–13.3). Readability scores did not differ across Cochrane Review Groups. SAs had a higher readability index than the corresponding PLSs (median = 16.6, 95% CI = 16.4–16.8). Regarding linguistic characteristics, PLSs were shorter than SAs, with less use of analytical tone, but more use of a positive emotional tone and authenticity. Overall, the ‘Unclear’ category of conclusiveness was the most common among all PLSs. Also, PLSs with ‘No evidence’ conclusions were the shortest and had the lowest SMOG index.

**Conclusion:**

PLSs of Cochrane systematic reviews of oncological interventions have low readability and most give unclear conclusions about the efficacy of interventions. PLSs should be simplified so that patients and their families can benefit from appropriate health information on evidence synthesis. Further research is needed into reasons for unclear language to describe evidence from oncology trials.

## Graphical abstract

Plain language summaries (PLSs) of Cochrane systematic reviews of oncological interventions have low readability and most give unclear conclusions about the efficacyof interventions. PLSs should be simplified so that patients and their families can benefit from appropriate health information on evidence synthesis.

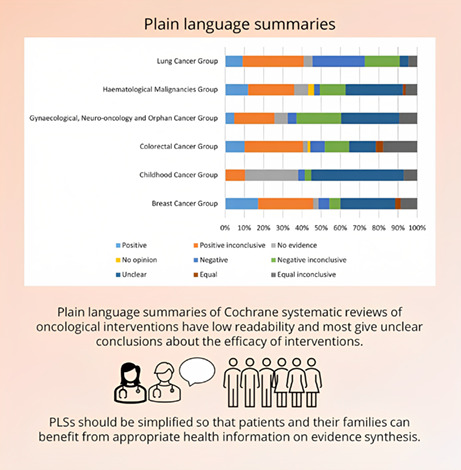

## 1 INTRODUCTION

Health literacy is defined as the degree to which individuals have the capacity to obtain, process and understand basic health information and services needed to make appropriate health decisions.^1^ Its importance has been confirmed through numerous studies, not only related to the health care of individuals but also in public health care system.^2^, ^3^, ^4^, ^5^

In the field of oncology, it is especially important for patients to have high level of health literacy, as well as the access to health information presented in a way understandable to the lay public. Cancer treatment options have very much advanced from conventional treatment approaches, and patients and their families are taking more active roles in treatment decisions.^6^ Once faced with a life‐changing diagnosis of cancer, most of them tend to seek further information from the Internet, forums, acquaintances, other patients, social support groups, articles, books and other sources.^7^, ^8^ However, studies have shown that nearly half of the patients with cancer have difficulty understanding information about the treatment for their particular type of cancer.^9^ Moreover, the readability of cancer information on the Internet is too demanding and most of the information does not meet the criteria for readability and understandability by lay population.^10^, ^11^, ^12^, ^13^, ^14^ Despite the evidence that the readability levels regarding oncology information for patients are unacceptably high, the readability of newly created materials from professional societies has not improved to an adequate level and there is still room for advancement in all oncology fields, across information providers.^12^, ^13^

One of the organizations that aims to improve the quality of health information for the public is Cochrane, whose Database of Systematic Reviews has emerged as one of the most trustworthy sources for the effectiveness of health interventions, due to its high‐quality systematic reviews.^15^ As a way of presenting the results and conclusions of systematic reviews, Cochrane Systematic Reviews include not only a scientific abstract (SA), but also a plain language summary (PLS).^16^

Plain language summaries are intended as a form of presenting the information from systematic reviews to the lay public and should be written in a way understandable to most readers.^16^ The level of readability and comprehension of the text can be influenced by different factors: emotional charge of the text, text characteristics, subjective expressions of attitudes or format of the presentation.^17^, ^18^, ^19^, ^20^, ^21^, ^22^ PLSs are written by the review authors.^18^ To ensure that PLSs are written in a standard format, the Cochrane has created the Standards for the Reporting of Plain Language Summaries in New Cochrane Intervention Reviews (PLEACS).^23^ It has been shown that these standards are often not followed.^24^ To improve the quality of the PLSs, several studies explored the influence of the factors such as the use of infographics,^25^ numerical presentation and framing of the direction of the results,^17^ as well as different summary formats.^26^ It was shown that there is still an effort to be made to produce content and format that will meet the need of health care users.^26^ PLSs and other sources of oncology information for lay public have been analysed in various ways.^17^, ^24^, ^25^, ^26^ Cochrane Systematic Review's PLSs of oncology interventions are an important format of reliable and understandable information to cancer patients and their families in their decision‐making, but their characteristics have not been studied. The aim of this study was to assess the language characteristics of PLS of Cochrane systematic reviews of oncology interventions in comparison with corresponding SAs.

## 2 METHODS

### 2.1 Study design and data sources

In this cross‐sectional study, we analysed the linguistic characteristics of PLSs and SAs of Cochrane systematic reviews of oncology interventions. Both types of summaries are written by review authors.

### 2.2 Inclusion and exclusion criteria

We included all PLSs and corresponding SAs available in the Cochrane Library up to February 2019. Summaries that were not intervention studies were excluded. We included abstracts from Cochrane Review Groups clearly focussed on oncology: Breast Cancer; Childhood Cancer; Colorectal Cancer; Gynaecological, Neuro‐oncology and Orphan Cancer; Haematological Malignancies; and Lung Cancer. These groups were taken as a proxy for different clinical types of cancer. Although there are systematic reviews in other Cochrane groups which can be placed in the field of oncology, we included summaries from groups that explicitly and only work on oncological systematic reviews.

Two authors (J.Š. and I.B.) analysed all included PLSs and corresponding SAs, discussing each abstract separately.

### 2.3 Data extraction

Data extraction spreadsheet was tested by two authors (J.Š. and I.B.). One author extracted the data, and the other one independently reviewed the data in a 10% random sample of PLSs and corresponding SAs. Then, it was checked whether the entry in the table was correct. The third author (A.M.) checked the entries from the extraction of the two authors in a sample of 20 PLSs and SAs at the beginning of the study. Interobserver agreement was high (kappa range 0.80–1.00, 95% confidence interval [CI] = 0.84–1.00). We resolved the differences in rating through the discussion with the third author (A.M.) before full data extraction. The extracted data included the name of the Cochrane Review Group, number of authors, year of publication, readability score, linguistic characteristics and categories of conclusiveness.

### 2.4 Simple measure of Gobbledygook index

The readability of summary formats in English was assessed using the Simple Measure of Gobbledygook (SMOG) index.^27^ SMOG index assesses the readability of certain content by counting polysyllabic words, and the result is presented as the number of years of education required to understand a given text.^28^ It is considered to be suitable for health information.^27^, ^28^ SMOG index for PLSs in English was calculated using an online tool: https://readable.com/.

Regarding SMOG index interpretation, the official recommendation of American Medical Association and National Health Institute is that health information should be written at the reading level of the sixth grade in the US education system.^29^, ^30^, ^31^

### 2.5 Linguistic characteristics

Plain language summaries and corresponding scientific summaries were analysed regarding their linguistic characteristics. To analyse the number of words and the length of the sentences, we used the Linguistic Inquiry and Word Count—LIWC (www.liwc.app/demo).^32^ This analysis is dictionary‐based and categorizes text words into four main variables: analytical, clout, authenticity and emotionality share in the tone of the text. These variables are presented as the percentage of words from the text in a particular category. Analytical thinking category is based on recognizing words associated with logic or with connecting concepts and putting them into a relationship. Greater use of words related to analytical thinking is related to cognitive complexity and to abstract thinking.^33^ Clout speech is a variable that refers to the use of terms that denote self‐confidence, leadership or social status. A higher proportion of such words suggest that the author speaks from a position of expertise and certainty in what is stated, and a lower proportion suggests a style of presenting information that is humbler.^34^ Authenticity is determined by the percentage of words related to personality, such as the use of personal nouns in the first person (‘I’, ‘my’ and ‘mine’), present tense and relative adverbs (near, now). Use of these words is connected to a writing that is more personal and honest.^35^, ^36^ Emotional share relates to how positive the tone is according to the words used. A score of 100 in emotional tone would mean the tone is maximally positive; a score of 50 means an even balance of positive and negative emotion words.^37^

### 2.6 Conclusiveness

Conclusiveness of statements about efficacy and safety was categorized into nine categories^39^:

1. Positive—there is (moderate/high‐quality) evidence of effectiveness/safety, that is the drug was proven effective/safe,
2. Positive inconclusive (there is evidence of effectiveness/safety, yet it is of a low quality/inconclusive or authors state that more research is required),
3. No evidence (there is no evidence from randomized controlled trials [RCTs] because the literature search did not result in any eligible studies, i.e. empty reviews),
4. No opinion (authors provided no opinion),
5. Negative—there is (moderate/high‐quality) evidence of no effect or evidence of harm (ineffectiveness/harmful) or authors advised against the intervention/comparison or it is not recommended,
6. Negative inconclusive (there is evidence of ineffectiveness/harm [evidence show that there was no effect or the intervention was not safe] or authors advised against the intervention/comparison or it is not recommended; yet evidence is of a low quality/ inconclusive or authors state that more research is required),
7. Unclear, more research is needed (authors state that more research is required),
8. Equal—analysed interventions were of equal effectiveness/safety, and
9. Equal inconclusive—interventions are equally effective/safe; yet evidence is of a low quality/inconclusive, or authors state that more research is required.^38^

### 2.7 Statistical analysis

The number of PLSs per Cochrane Review Group and categories of conclusiveness were described as frequencies and percentages. The number of words and sentences, readability score and linguistic variables were described as median with interquartile range (IQR) or 95% CI, due to the uneven distribution of data, as determined by Kolmogorov Smirnov test. For comparison of summaries of different oncology groups, we used Kruskal–Wallis test and Conover Iman post hoc test. Conover Iman test was used for post hoc comparisons to account for multiple comparisons. All analysis was done in JASP version 0.12.1.0. (JASP Team, 2020, https://jasp‐stats.org/JASP).

## 3 RESULTS

**FIGURE 1:**
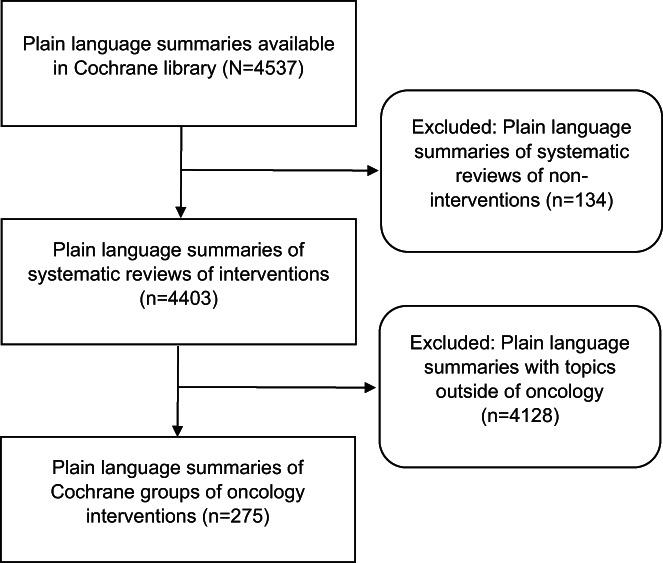
Selection of plain language summaries for the study.

In total, we collected 275 Cochrane PLSs and corresponding SAs of systematic reviews of oncology interventions (Figure 1). The median number of authors for systematic review was 5 (IQR = 4–6). The analysis of the summaries in different Cochrane Cancer Review Groups showed that the fewest number of authors was in the Childhood Cancer Group and Colorectal Cancer Group (Table 1).

**TABLE 1:** Number of authors, text lengths and readability index (median, 95% confidence interval) of plain language summaries and scientific abstracts across six Cochrane Cancer Review Groups (*N* = 275).

	Breast Cancer Group (*n* = 35)	Childhood Cancer Group (*n* = 29)	Colorectal Cancer Group (*n* = 79)	Gynaecological, Neuro‐oncology and Orphan Cancer Group (*n* = 43)	Haematological Malignancies Group (*n* = 67)	Lung Cancer Group (*n* = 22)	Total	*p* ^a^
Number of review authors	5 (4–6)	5 (4–5)^b^ ^,^ ^c^	5 (4–5)^c^	6 (5–6)	6 (5–6)	5 (4–5.5)	5 (5–5)	0.002
Plain language summaries:								
Text length (No. words)	400 (367–433)	314 (241–393)^d^	221 (166–268)^d^ ^,^ ^e^	410 (372–440)^e^	475 (427–526)^e^	279 (200–376)^c^ ^,^ ^d^	364 (339–388)	<0.001
Readability^f^	12.8 (11.6–13.1)	12.5 (11.8–13.1)	13.3 (12.6–13.4)	13.3 (12.7–13.6)	13.0 (12.7–13.2)	12.4 (11.8–13.0)	13.0 (12.8–13.3)	0.053
Scientific abstracts:								
Text length (No. words)	575 (450–723)^b^ ^,^ ^c^ ^,^ ^e^	643 (538–715)^b^	458 (420–492)	715 (642–765)^b^	714 (626–793)^b^	604 (497–694)^b^ ^,^ ^c^	604 (566–653)	<0.001
Readability^f^	16.5 (16.0–17.1)	16.4 (15.4–16.5)	16.4 (16.0–16.8)	16.9 (16.2–17.4)	16.7 (16.4–16.8)	16.7 (15.7–17.3)	16.6 (16.4–16.8)	0.462

^a^ Kruskal–Wallis test and Conover Iman post hoc test.

^b^ Significantly different from Colorectal Cancer Group.

^c^ Significantly different from Gynaecological, Neuro‐oncology and Orphan Cancer Groups.

^d^ Significantly different from Breast Cancer Group.

^e^ Significantly different from Childhood Cancer Group.

^f^ Readability was measured as a SMOG index.^27^, ^28^ Higher scores indicated lower readability.

### 3.1 Readability analysis

In general, PLSs were shorter and easier to read than SAs. SMOG index for PLSs was double the recommended 6 years of education (median = 13.0, 95% CI = 12.8–13.3), compared to 16.6 (95% CI = 16.4–16.8) for SAs (*p* < 0.001; Wilcoxon nonparametric paired samples test). SAs were also significantly longer than PLSs: median word count 604 (95% CI = 566–653) versus 364 (95% CI = 339–388), respectively (*p* < 0.001; Wilcoxon nonparametric paired samples test).

Readability scores did not differ among different Cochrane Cancer Review Groups, both for PLSs and for SAs (Table 1). The Groups differed in the text length of the summaries, with both PLSs and SAs being the shortest for the Colorectal Cancer Group (Table 1).

### 3.2 Linguistic characteristics

The use of the analytical tone was most prominent in both PLSs and SAs (Table 2). Half of the words in both PLSs and SAs were categorized as clout speaking. Authentic and emotional tones were used less frequently in both types of summaries. In general, PLSs used language with significantly more authenticity and emotional tones, but fewer words related to analytical expressions than SAs.

**TABLE 2:** Linguistic characteristics (median, 95% confidence interval) of Cochrane systematic review plain language summaries and corresponding scientific abstracts.

	PLS	SAs	*p*
Analytic	95.5 (95.0–95.8)	96.6 (96.4–97.0)	<0.001
Clout	50.0 (47.7–51.8)	50.7 (48.8–52.4)	0.181
Authentic	24.1 (21.8–27.2)	17.9 (17.0–19.5)	<0.001
Emotional tone	22.1 (18.0–25.8)	20.4 (17.5–23.4)	0.014

*Note*: Linguistic characteristics of the text were measured using dictionary‐based text word categorizations by the Linguistic Inquiry and Word Count—LIWC.^32^ The variables are presented as the percentage of words from the text in a particular category. Statistics: Wilcoxon nonparametric paired samples test.

Abbreviations: PLS, plain language summary; SA, scientific abstract.

With regard to different Cochrane Cancer Review Groups, there was no statistically significant difference in the use of the analytical tone in PLSs (Table 3). The clout tone was more dominant in PLSs from Gynaecological, Neuro‐oncology and Orphan Cancer Groups. In the Childhood Cancer Group, we observed the least use of emotional tone, while the authentic tone was least used in Haematological Malignancies Group (Table 3).

**TABLE 3:** Linguistic characteristics of plain language summaries (median, 95% confidence interval) across six Cochrane Cancer Review Groups (*n* = 275).

	Breast Cancer Group (*n* = 35)	Childhood Cancer Group (*n* = 29)	Colorectal Cancer Group (*n* = 79)	Gynaecological, Neuro‐oncology and Orphan Cancer Group (*n* = 43)	Haematological Malignancies Group (*n* = 67)	Lung Cancer Group (*n* = 22)	Total	*p*
Analytical tone	95.4 (94.8–96.2)	95.5 (93.8–96.4)	96.5 (95.5–96.8)	93.6 (91.5–94.8)	95.8 (94.6–96.4)	93.8 (90.9–95.5)	95.5 (95.0–95.8)	0.009
Clout tone	50.8 (45.4–57.8)^a^	50.0 (43.3–55.4)^a^	43.8 (40.4–47.5)	55.3 (51.1–56.2)^a^	49.0 (46.5–53.8)^a^ ^,^ ^b^	52.3 (46.8–53.6)^a^	50.0 (47.7–51.8)	<0.001
Authentic tone	23.3(17.0–31.3^c^	25.4 (20.5–30.7)^c^	27.3 (19.0–30.3)^c^	27.2 (21.8–31.2)^c^	19.8 (15.0–22.8)	34.8 (18.9–40.5)^c^	24.1 (21.8–27.2)	0.005
Emotional tone	28.1 (17.5–39.3)^c^, ^d^	10.5 (3.7–25.8)	25.8 (16.8–25.8)^c^, ^d^	18.0 (12.9–30.7)	16.7 (11.2–19.9)	25.8 (13.7–30.1)^,^ ^c^, ^d^	22.1 (18.0–25.8)	0.004

*Note*: Linguistic characteristics of the text were measured using dictionary‐based text word categorizations by the Linguistic Inquiry and Word Count—LIWC.^32^ The variables are presented as the percentage of words from the text in a particular category. Statistics: Kruskal–Wallis test and Conover Iman post hoc test.

^a^ Significantly different from Colorectal Cancer Group.

^b^ Significantly different from Gynaecological, Neuro‐oncology and Orphan Cancer Groups.

^c^ Significantly different from Haematological Group.

^d^ Significantly different from Childhood Cancer Group.

As in PLSs, the predominant use of the clout tone was found in SAs from Gynaecological, Neuro‐oncology and Orphan Cancer Groups. The analytical tone was predominant in SAs in Childhood Cancer Group. SAs from the Lung Cancer Group had the greatest proportion of words related to authenticity, as well as the greatest use of emotional tone (Table 4).

**TABLE 4:** Linguistic characteristics of scientific abstracts (median, 95% confidence interval) across six Cochrane Cancer Review Groups (*n* = 275).

	Breast Cancer Group (*n* = 35)	Childhood Cancer Group (*n* = 29)	Colorectal Cancer Group (*n* = 79)	Gynaecological, Neuro‐oncology and Orphan Cancer Group (*n* = 43)	Haematological Malignancies Group (*n* = 67)	Lung Cancer Group (*n* = 22)	Total	*p*
Analytical tone	96.8 (96.0–97.3)	97.4 (97.1–97.6)	96.3 (95.9–96.6)^a^	96.4 (95.4–96.6)^a^	96.8 (96.1–97.1)^a^	97.1 (95.8–97.6)^b^	96.6 (96.4–97.0)	<0.001
Clout tone	53.5 (48.4–55.9)^b^	51.6 (44.6–54.2)^b^	45.9 (43.1–47.8)	55.0 (52.4–56.4)^b^	51.9 (48.6–53.8)^b^, ^c^	49.7 (46.7–57.6)^b^	50.7 (48.8–52.4)	<0.001
Authentic tone	17.1 (14.9–19.8)	19.7 (15.1–26.8)^a^	20.0 (16.9–20.0)^a^	18.4 (15.7–21.5)^a^	14.6 (13.8–17.2)	23.7 (13.7–29.5)^a^	17.9 (17.0–19.5)	<0.020
Emotional tone	25.8 (17.3–37.2)^a^	17.3 (11.5–22.2)	25.8 (17.6–25.8)^a^	18.2 (10.8–24.0)	11.6 (9.5–16.6)	26.1 (14.0–37.8)^a^	20.4 (17.5–23.4)	<0.001

*Note*: Linguistic characteristics of the text were measured using dictionary‐based text word categorizations by the Linguistic Inquiry and Word Count—LIWC.^32^ The variables are presented as the percentage of words from the text in a particular category. Statistics: Kruskal–Wallis test and Conover Iman post hoc test.

^a^ Significantly different from Childhood Cancer Group.

^b^ Significantly different from Colorectal Cancer Group.

^c^ Significantly different from Gynaecological, Neuro‐oncology and Orphan Cancer Groups.

### 3.3 Conclusiveness

**FIGURE 2:**
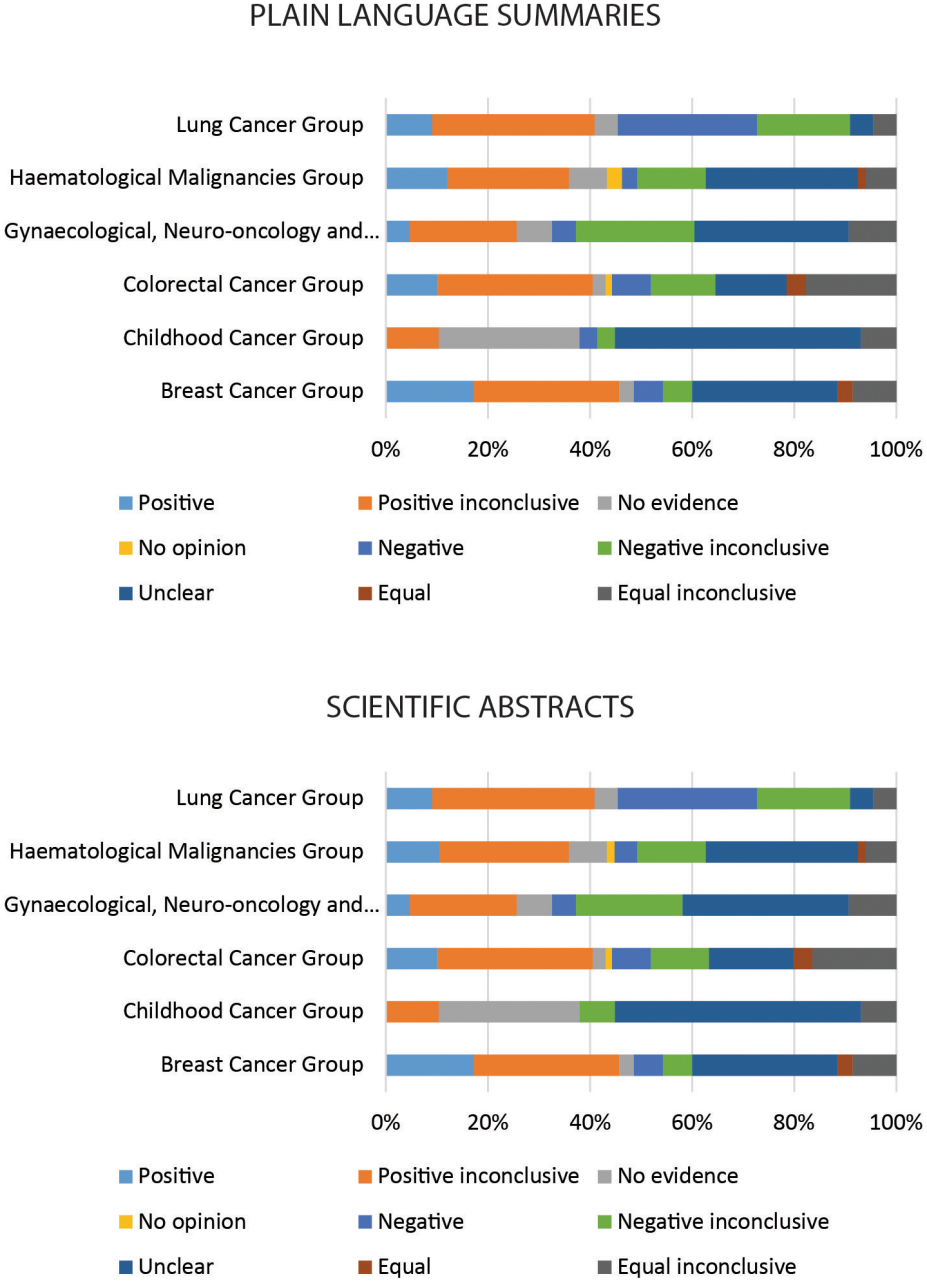
Categories of conclusiveness in plain language summaries and scientific abstracts of systematic reviews from Cochrane cancer review groups.

Categories of conclusiveness differed between summaries from different Cochrane Review Groups. PLSs from the Lung Cancer Group there had the greatest proportion of negative conclusion compared with the other groups. In Childhood Cancer Group, we found no PLSs with positive conclusions (Figure 2; Table S2). In Gynaecological, Neuro‐oncology and Orphan Cancer Group and Breast Cancer Group, we found no summaries where the authors did not provide an opinion (Figure 2; Table S2). SAs followed a similar pattern (Figure 2; Table S1). Seven (2.5%) out of 275 pairs of SAs and PLSs did not agree in conclusiveness category (one in Childhood Cancer Group, two in Colorectal Cancer Group, one in Gynaecological, Neuro‐oncology and Orphan Cancer Group, two in Haematological Malignancies Group and one in Lung Cancer Group).

We also analysed the readability of PLSs regarding their conclusion. Among PLSs, those summaries that provided ‘No evidence’ conclusion were significantly easier to read than the summaries with other conclusions (Table S3). There was no statistically significant difference in the readability of SAs with different conclusions (Table S4). Regarding the readability of PLSs versus SAs with different conclusiveness, seven pairs did not match in conclusiveness, so it was not possible to analyse these differences on the whole sample.

Plain language summaries with a ‘Positive’ conclusion had significantly greater use of analytic tone. There was no significant difference in other linguistic characteristics, that is use of clout, authentic and emotional tones. Furthermore, there was no statistically significant difference for the length of the summaries with different conclusions (Table S5). In contrast, SAs with ‘No evidence’ or ‘Negative’ conclusions were significantly shorter than the abstracts with other conclusions. There was no significant difference in the linguistic characteristics of SAs with different conclusion types (Table S6).

## 4 DISCUSSION

In this study of a cohort of PLSs and corresponding SAs from six Cochrane Review Groups in oncology, PLSs were easier to read than the corresponding SAs, but the required literacy level was still too high for a general population. PLSs were shorter than SAs and had less use of analytical tone, with more use of authenticity and emotional tones.

These results should be interpreted with several limitations in mind. First, we used only the Cochrane Library as a source of summaries for scientific papers. However, summaries from the Cochrane Library had the same format of presenting health information, making them comparable. Second, we only analysed the summaries in English, since it was the only common language for the summaries of oncology.

On average, 13 years of education was needed to read a PLS for the systematic reviews in oncology from our study. The American Medical Association and National Health Institute recommended that the reading grade for the texts with health information intended for the lay public should be 6,^30^, ^31^ meaning that 6 years of education are needed for the comprehension of a given text. When writing a PLS for a scientific article, it is not easy to achieve that reading level because complex scientific expressions need to be simplified; at the same time, the information must be translated to the reader in an accurate way. Our study showed that there was no significant difference in readability across Cochrane Review Groups related to oncology, so there is a clear need for an overall improvement of PLSs in the field of oncology.

Improving PLSs is only a part of the solution—we must bear in mind the current state of health literacy in the general public, as it has been shown that about a third of US citizens have low health literacy.^39^, ^40^ Moreover, it has been demonstrated that lay public rarely uses health information based on evidence‐based medicine, like scientific articles or websites of professional organizations.^26^, ^27^, ^41^ Instead, most of them tend to use commercial websites.^26^, ^27^

Both the PLSs and SAs are written by the same authors, who are scientists and are not trained in writing for nonscientific audiences.^42^ It has been shown that press releases for Cochrane systematic reviews, written by communication specialists, have a more engaging language in comparison with PLSs and SAs.^18^ A part of the solution to make a bridge between low health literacy of the lay public on one side and complex health information on the other side would be a coordinated work of health journalists alongside with oncology professionals. The need for more input from professional communicators is supported by our finding that a small number of PLSs and SAs pairs differed in the category of conclusiveness, although they were written by the same authors.

Possible option for translating SAs into health‐related material for lay public could be use of artificial intelligence‐using algorithms that will be instructed to achieve the recommended readability levels. Different media channels available to the public, like radio, TV or social media networks could be used for consistent communication, although the simplicity of sharing information in these ways could be a double‐edged sword because it is as easy to share a misinformation as it is to share correct information.^43^ The importance of health literacy and its impact on social and economic determinants of health has already been recognized by some government agencies, which led to forming national plans for improving health literacy.^44^, ^45^, ^46^

Regarding the linguistic characteristics of the textual summaries of Cochrane systematic reviews, we found them to be similar in both PLSs and SAs: the most of the words were related to analytical tone, about half of the words were related to clout speaking, while approximately one quarter was categorized as authentic and emotional speaking. This similarity can be explained by the same authorship of the two types of texts. There still were some differences between PLSs and SAs: in general, PLSs were shorter and used less analytical and more authentic and emotional tones compared with SAs. As the aim of an SA is an accurate presentation of facts, it can explain the higher analytical tone compared with a PLS. The analytical tone relates to heightened abstract thinking and cognitive complexity.^33^ On the contrary, the use of authentic tone is connected to a writing that is personal, honest and less filtered,^36^, ^37^ which suits PLSs and their intention to bring the subject closer to the lay public. The emotional tone relates to how positive the tone is.^37^ Apparently, scientists tend to look on the bright side of research results in general,^47^ and it appears that, when writing PLSs, authors tend to write them in a more positive tone, maybe due to their inexperience as writers for general public engaging materials.

Most of the systematic reviews gave unclear conclusions about the efficacy of intervention and most of them concluded that further evidence is needed for definitive decision. This can be explained by the lack of sufficient evidence for new treatments. For the development of a new cancer drug, the essential part of evaluating the efficacy of a new therapeutic option are clinical trials. However, it has been estimated that less than 5% of adult cancer patients enrol in cancer clinical trials.^48^, ^49^ On one hand, there is a need for more clinical trials, but on the other hand, small number of patients participate in them, even though 70% of cancer patients in the United States seem to be willing to participate in clinical trials.^50^ Unclear conclusion at the end of the summary can be the source of confusion for the lay public. To prevent that, authors could state that the summary does not provide a clear answer and suggest consultation with a medical oncologist, especially because every treatment decision should be made upon a thorough assessment of the oncologic value of treatment.

Childhood Cancer Review Group was the one where we found no summaries with positive conclusions. The rarity of childhood cancer, combined with safety and regulatory requirements, makes it very demanding to conduct RCTs.^51^ We still mostly rely on indirect evidence from adult population, early phase clinical trials and case series, where it is challenging to make conclusions on the effectiveness and safety of an intervention.^52^ More randomized evidence and individual patient data meta‐analyses are needed to increase certainty and precision in the care of paediatric cancer patients.

Plain language summaries from Breast Cancer Review Group were the PLSs that had the most positive conclusions. Breast cancer is the most common cancer in women.^53^ Over the last two decades, we have witnessed an enhancement of the conventional oncology treatments with new options such as immunotherapy, conjugated antibodies and targeting other metabolic pathways.^53^ Conducting randomized trials is relatively simpler for this cancer than for other, less common types of cancer; and there is also a strong commercial interest for creating new therapies.

On the other side, PLSs from the Lung Cancer Review Group had the greatest proportion of negative conclusions. As mentioned before, new therapeutic options are available, especially for nonsmall lung cancer.^54^ Yet, despite these options and new clinical trials, lung cancer remains a significant challenge and contributes to most cases of cancer‐related death worldwide.^55^ The part of the problem, aside from tumour resistance to therapeutic options, is that lung cancer is mostly diagnosed in stages III and IV,^56^ unlike breast cancer, where screening programme shifted to the earlier stage at the time of diagnosis.^57^

We found that the summaries that provided ‘No evidence’ conclusion were significantly easier to read than the summaries with other conclusions. This is easy to explain since there was nothing to discuss or further elaborate in a summary if there was no evidence. However, we found no statistically significant difference in the readability of the PLSs with different conclusions, so the conclusion about the efficacy of given therapy or intervention does not seem to influence the readability of a PLS. This finding is in line with previous studies^58^, ^59^ that have shown that scientific literature is becoming harder to read in general and that scientists tend to use scientific jargon when writing, so further simplification for PLSs is still a translational challenge.

## 5 CONCLUSION

Plain language summaries of Cochrane systematic reviews of oncology interventions have low readability and most give unclear conclusion about the effectiveness of therapy and procedures. To enhance the quality of the PLSs, authors and Cochrane Review Groups should be more attentive to follow the standards for writing summaries intended for general public. An input from the professionals who write public engaging materials, such as journalists, may help create health information material that will satisfy different health users yet provide sufficient scientific information and dissemination of evidence. Further research should focus on the improvement of PLSs and deeper analysis of reasons for unclear evidence in oncology trials.

## AUTHOR CONTRIBUTIONS

**Jelena Šuto:** Investigation (equal); writing – original draft (lead). **Ivan Buljan:** Methodology (equal); writing – review and editing (equal). **Ana Marušić:** Supervision (lead); writing – review and editing (equal).

## FUNDING INFORMATION

This study was funded by the Croatian Science Foundation (‘Professionalism in Health—Decision making in practice and research, ProDeM’) under Grant agreement No. IP‐2019‐04‐4882. The funder had no role in the design of this study, its execution, analyses, interpretation of the data or decision to submit results.

## CONFLICT OF INTEREST STATEMENT

The authors declare no conflict of interest except for the membership in Cochrane.

## Supporting information


Table S1.
Table S2.
Table S3.
Table S4.
Table S5.
Table S6.

## Data Availability

The data that support the findings of this study are available from the corresponding author upon reasonable request.
