# Supplementary material for: Linguistic analysis of plain language summaries and corresponding scientific summaries of Cochrane systematic reviews about oncology interventions
Source: Cancer Med. 2023 Mar 23;12(9):10950–60. doi: 10.1002/cam4.5825 (PMC10225178; doi:10.1002/cam4.5825)
Supplement: Supplementary file 1 — Table S1. Table S2. Table S3. Table S4. Table S5. Table S6. [file CAM4-12-10950-s001.docx]

**Supplementary Document**

Full linguistic analysis, Tables S1-S6.

**Table S1.** Distribution of conclusiveness categories across six groups of Cochrane Cancer review in Cochrane scientific abstracts (N=275)*

| **Category** | **Breast**  **Cancer**  **Group**  **(N=35)** | **Childhood Cancer**  **Group**  **(N=29)** | **Colorectal Cancer**  **Group**  **(N=79)** | **Gynaecological, Neuro-oncology and Orphan Cancer Group (N=43)** | **Haematological Malignancies**  **Group**  **(N=67)** | **Lung**  **Cancer**  **Group**  **(N=22)** | **Total**  **(N=275)** |
| --- | --- | --- | --- | --- | --- | --- | --- |
| **Positive** | 6 (17.1%) | 0 (0.0%) | 8 (10.1%) | 2 (4.5%) | 7 (10.4%) | 2 (9.1%) | 25 (9.1%) |
| **Positive inconclusive** | 10 (28.6%) | 3 (10.3%) | 24 (30.4%) | 9 (20.9%) | 17 (25.4%) | 7 (31.8%) | 67 (24.5%) |
| **No evidence** | 1 (2.9%) | 8 (27.6%) | 2 (2.5%) | 3 (7.0%) | 5 (7.5%) | 1 (4.5%) | 20 (7.3%) |
| **No opinion** | 0 (0.0%) | 0 (0.0%) | 1 (1.3%) | 0 (0.0%) | 1 (1.5%) | 0 (0.0%) | 2 (0.7%) |
| **Negative** | 2 (5.7%) | 0 (0.0%) | 6 (7.659%) | 2 (4.6%) | 3 (4.5%) | 6 (27.3%) | 19 (6.9%) |
| **Negative inconclusive** | 2 (5.7%) | 2 (6.9%) | 9 (11.4%) | 9 (20.9%) | 9 (13.4%) | 4 (18.2%) | 35 (12.7%) |
| **Unclear** | 10 (28.6%) | 14 (48.3%) | 13 (16.5%) | 14 (32.6%) | 20 (29.8%) | 1 (4.5%) | 72 (26.2%) |
| **Equal** | 1 (2.9%) | 0 (0.0%) | 3 (3.8%) | 0 (0.0%) | 1 (1.5%) | 0 (0.0%) | 5 (1.8%) |
| **Equal inconclusive** | 3 (8.6%) | 2 (6.9%) | 13 (16.5%) | 4 (9.3%) | 4 (56.0%) | 1 (4.5%) | 27 (9.8%) |

*Column percentages; they may not add up to 100 because of rounding.

**Table S2.** Distribution of conclusiveness categories across six groups of Cochrane Cancer review in Cochrane plain language summaries (N=275)

| **Category** | **Breast Cancer Group (N=35)** | **Childhood Cancer Group (N=29)** | | **Colorectal Cancer**  **Group**  **(N=79)** | | **Gynaecological, Neuro-oncology and Orphan Cancer Group (N=43)** | | **Haematological Malignancies Group**  **(N=67)** | **Lung Cancer Group (N=22)** | **Total (N=275)** |
| --- | --- | --- | --- | --- | --- | --- | --- | --- | --- | --- |
| **Positive** | 6 (17.1%) | | 0 (0.0%) | | 8 (10.1%) | 2 (4.7%) | 8 (11.9%) | | 2 (9.1%) | 26 (9.5%) |
| **Positive inconclusive** | 10 (28.6%) | | 3 (10.3%) | | 24 (30.4%) | 9 (20.9%) | 16 (23.9%) | | 7 (31.8%) | 69 (25.1%) |
| **No evidence** | 1 (2.9%) | | 8 (27.6%) | | 2 (2.5%) | 3 (7.0%) | 5 (7.5%) | | 1 (4.6%) | 20 (7.3%) |
| **No opinion** | 0 (0.0%) | | 0 (0.0%) | | 1 (1.3%) | 0 (0.0%) | 2 (3.0%) | | 0 (0.0%) | 3 (1.1%) |
| **Negative** | 2 (5.7%) | | 1 (3.5%) | | 6 (7.6%) | 2 (4.7%) | 2 (3.0%) | | 6 (27.3%) | 19 (6.9%) |
| **Negative inconclusive** | 2 (5.7%) | | 1 (3.5%) | | 10 (12.7%) | 10 (23.3%) | 9 (13.4%) | | 4 (18.2%) | 36 (13.1%) |
| **Unclear** | 10 (28.6%) | | 14 (48.3%) | | 11 (13.9%) | 13 (30.2%) | 20 (29.9%) | | 1 (4.6%) | 69 (25.1%) |
| **Equal** | 1 (2.9%) | | 0 (0.0%) | | 3 (3.8%) | 0 (0.0%) | 1 (1.5%) | | 0 (0.0%) | 5 (1.8%) |
| **Equal inconclusive** | 3 (8.6%) | | 2 (6.9%) | | 14 (17.7%) | 4 (9.3%) | 4 (6.0%) | | 1 (4.6%) | 28 (10.2%) |

*Column percentages; they may not add up to 100 because of rounding.

**Table S3.** Comparison of readability characteristics (median, 95% confidence interval) across plain language summaries with different conclusions

|  | **Positive (n=26)** | **Positive inconclusive (n=69)** | **No evidence (n=20)** | **Negative (n=19)** | **Negative inconclusive (n=36)** | **Unclear (n=69)** | **Equal inconclusive (n=28)** | ***P**** |
| --- | --- | --- | --- | --- | --- | --- | --- | --- |
| **SMOG-index** | 13.6  (12.4-14.3)†‡ | 12.8  (12.1-13.0) | 12.0  (11.3-12.4) | 13.5  (12.5-14.1)† | 12.6  (12.1 -13.7)† | 12.9  (12.1-12.9)† | 13.4  (13.2-13.9)† | 0.009 |

Excluded: “No opinion” and “Equal” categories due to the low sample size.

*Kruskal Wallis test and Conover Iman post-hoc test.

†Significantly different from “No evidence”.

‡Significantly different from “Positive inconclusive”.

**Table S4.** Comparison of readability characteristics (median, 95% confidence interval) across scientific abstracts with different conclusions

|  | **Positive (n=25)** | **Positive inconclusive (n=70)** | **No**  **evidence (n=20)** | **Negative**  **(n=19)** | **Negative**  **inconclusive**  **(n=35)** | **Unclear**  **(n=72)** | **Equal inconclusive (n=27)** | ***P**** |
| --- | --- | --- | --- | --- | --- | --- | --- | --- |
| **SMOG index** | 16.7  (16.0-17.2) | 16.5  (16.2-17.0) | 16.2  (15.6-16.8) | 16.3  (15.8-17.2) | 16.6  (15.5-17.0) | 16.7  (16.3-16.9) | 16.5  (15.9-17.4) | 0.753 |

Excluded: “No opinion” and “Equal” categories due to the low sample size.

*Kruskal Wallis test and Conover Iman post-hoc test.

**Table S5**. Comparison of linguistic characteristics (median, 95% confidence interval) across plain language summaries with different conclusions

|  | **Positive (n=26)** | **Positive inconclusive (n=69)** | **No evidence (n=20)** | **Negative (n=19)** | **Negative inconclusive (n=36)** | **Unclear (n=69)** | **Equal inconclusive (n=28)** | ***P**** |
| --- | --- | --- | --- | --- | --- | --- | --- | --- |
| **Word count** | 384  (22.9-478) | 343  (313-386) | 382  (216-416) | 200  (150-281) | 374  (261-426) | 395  (351-414) | 353  (242-392) | 0.052 |
| **Analytic** | 97.4  (96.2 -98.2) | 95.3  (94.4-96.3)§ | 94.1  (91.4-95.8)§ | 95.0  (92.0-95.6)§ | 94.2  (93.4-95.4)§ | 95.6  (94.6-96.1)§ | 95.6  (93.2-96.9)§ | 0.008 |
| **Clout** | 46.6  (40.1-52.2) | 50.0  (43.0-52.8) | 52.4  (44.9-56.0) | 46.1  (32.8-48.7) | 49.7  (44.5-52.3) | 55.3  (47.0-57.3) | 47.6  (40.9-51.4) | 0.058 |
| **Authentic** | 25.5  (17.5-27.3) | 27.2  (19.0-30.1) | 19.4  (13.3-25.7) | 31.2  (17.1-37.2) | 21.2  (16.4-30.7) | 23.3  (20.2-27.0) | 27.5  (19.9-32.7) | 0.498 |
| **Emotional** | 25.8  (12.1-27.5) | 20.4  (15.4-25.8) | 25.8  (7.2-42.6) | 17.2  (8.7-31.2) | 22.8  (12.5-30.1) | 19.9  (13.6-25.8) | 25.8  (12.0-29.4) | 0.953 |

Excluded: “No opinion” and “Equal” categories due to the low sample size.

*Kruskal Wallis test and Conover Iman post-hoc test.

†Significantly different from “No evidence”.

‡ Significantly different from “Positive inconclusive”.

§Significantly different from “Positive”.

**Table S6.** Comparison of linguistic characteristics (median, 95% confidence interval) across scientific abstracts with different conclusions

|  | **Positive (n=25)** | **Positive inconclusive (n=70)** | **No evidence (n=20)** | **Negative (n=19)** | **Negative inconclusive (n=35)** | **Unclear (n=72)** | **Equal inconclusive (n=27)** | ***P**** |
| --- | --- | --- | --- | --- | --- | --- | --- | --- |
| **Word count** | 603  (407-746)†‡ | 692  (581-743)†‡ | 459  (346-527) | 425  (374-485) | 645  (526-696)†‡ | 633  (577-724)†‡ | 566  (437-670)‡ | <0.001 |
| **Analytic** | 97.0  (96.2-97.3) | 96.2  (95.6-97.0) | 96.4  (94.1- 97.3) | 95.6  (94.6-96.7) | 96.7  (96.1-97.3) | 96.8  (96.4-97.2) | 97.1  (96.2-97.2) | 0.164 |
| **Clout** | 48.8  (44.5-53.2) | 48.7  (46.0-52.4) | 52.2  (50.0- 55.6) | 47.4  (41.0-51.4) | 51.7  (45.5-54.8) | 53.1  (48.7-55.0) | 50.7  (47.2-52.6) | 0.293 |
| **Authentic** | 16.1  (12.8-23.2) | 20.0  (18.0-22.8) | 17.6  (14.1-19.2) | 23.5  (18.7-28.0) | 14.2  (11.8-17.3) | 17.4  (14.7-22.0) | 19.2  (14.1-22.5) | 0.050 |
| **Emotional** | 25.8  (14.2-31.4) | 22.1  (11.8-27.7) | 27.4  (16.5-39.1) | 20.5  (11.5-40.6) | 19.4  (12.2-29.0) | 17.3  (14.4-20.8) | 22.2  (11.5-23.4) | 0.364 |

Excluded: “No opinion” and “Equal” categories due to the low sample size.

*Kruskal Wallis test and Conover Iman post-hoc test.

†Significantly different from “No evidence”.

‡ Significantly different from “Negative”.
